# The Critical Help-Seeking Role of Family, Friends, and Neighbors in the Lives of Elder Abuse Victims

**DOI:** 10.1093/geroni/igaa057.2143

**Published:** 2020-12-16

**Authors:** Marlene Stum, David Burnes

## Abstract

Elder abuse prevention and intervention is a complex puzzle. We focus on examining the typically invisible role, experience, and impact of nonabusing family, friends, and neighbors, or “concerned persons” in stopping elder abuse. Given the reality that most elder abuse goes unreported and unaddressed, it seems essential to understand if and how concerned persons can play a role in help-seeking for older victims, and to also understand the needs and issues faced by concerned persons as a consequence. First. Breckman presents evidence of the significant distress concerned person’s experience from knowing about elder abuse and trying to assist victims, and shares experience developing and implementing the first Elder Abuse Helpline for Concerned Persons in the U.S. Second, Fraga Dominguez et.al. present an important international perspective highlighting findings about concerned persons as users of a UK elder abuse helpline, their profile, the impact of helping, and variables relating to help-seeking. Third, Stum shares findings from a qualitative study of elder family financial exploitation related to what concerned family members were trying to accomplish by getting involved (motivating goals) and the resulting continuum of outcomes. Fourth, Kilaberia also explores the help-seeking experiences of concerned family members in elder family financial exploitation situations, specifically the range of tasks involved, and the impacts on the concerned family member’s individual health and well- The discussion led by Burnes will focus on understanding contributions of the research presented given the current state of the field, and offer suggestions for future research and intervention directions.

